# A Survey on the Availability and Accessibility of Psychoeducation Resources Within Child and Adolescent Mental Health Services (CAMHS) in Black Country Healthcare NHS Foundation Trust (BCHFT)

**DOI:** 10.1192/bjo.2026.11264

**Published:** 2026-06-30

**Authors:** Reka Ajay Sundhar, Bolutife Oyatokun, Oladipo Sowunmi, Muhammad Mujahid, Arif Khan

**Affiliations:** 1 Black Country Healthcare NHS Foundation Trust, Dudley, United Kingdom; 2 Black Country Healthcare NHS Foundation Trust, Walsall, United Kingdom; 3 Black Country Healthcare NHS Foundation Trust, Wolverhampton, United Kingdom

## Abstract

**Aims:**

Psycho education is a vital component of holistic and effective care within Child and Adolescent Mental Health Services (CAMHS). High-quality, accessible information enables young people and their families to better understand their mental health conditions and recommended treatments. This helps engagement, informed decision-making, improve clinical outcomes and patient safety. However, services face challenges in delivering consistent psychoeducation due to variability in available resources and clinician awareness. This Quality Improvement Project aimed to assess clinicians’ awareness, confidence, and use of psychoeducation resources within CAMHS in BCHFT and to develop a centralised system to support high-quality psychoeducation across the Trust.

**Methods:**

This project was developed using the PDSA quality-improvement framework. An anonymous self-report survey was distributed to clinicians across the four boroughs, consisting of 14 items: 11 Likert-scale questions and 3 open-ended prompts. The survey examined clinicians’ awareness of psychoeducation resources, confidence in delivering psychoeducation, frequency of use, and familiarity with local materials such as websites, books, apps, leaflets, workshops, and skills groups. Clinicians were also invited to propose ways to improve psychoeducation provision to guide us in the development of a centralised resource for each borough.

**Results:**

A total of 32 clinician responses were analysed. Most respondents (96%) recognisedthe importance of psychoeducation for recovery and relapse prevention, and 90.5% reported routinely offering it in consultations. However, notable knowledge gaps emerged: 37% were unaware or strongly unaware of the Trust’s Service Directory, and 25.5% were neutral or unaware of existing CAMHS psychoeducation workshops. Although 85% felt confident delivering psychoeducation, only 62% were satisfied with current provision. Verbal explanation and website signposting were used most frequently, followed by leaflets. Clinicians recommended improving access to resources through printed materials, shared drives, and Microsoft SharePoint, as well as increasing availability in waiting areas. Additional suggestions included materials on parental mental health, coping strategies, condition summaries, staff training, and a parent portal.

**Conclusion:**

The survey highlighted a wide range of psychoeducation resources used across CAMHS but revealed inconsistent awareness, with more experienced clinicians accessing a broader selection. These findings highlighted the need for a centralised approach to share resources. In response, a resource booklet has been developed for each locality and will be circulated to all clinicians, with plans for regular review. This project aims to strengthen clinician confidence, improve service quality, and enhance the wellbeing and safety of children and families accessing CAMHS.

